# Secondary sclerosing cholangitis in localized hepatobiliary tuberculosis simulating cholangiocarcinoma: a rare case report

**DOI:** 10.1186/s12876-017-0690-x

**Published:** 2017-11-28

**Authors:** Aleena Jain, Rachana Chaturvedi, Chetan Kantharia, Amita Joshi, Mangesh Londhe, Mayura Kekan

**Affiliations:** 1 Department of Pathology, Seth GSMC & KEMH, Parel, Mumbai, India; 2 Department of Pathology, Seth GSMC & KEMH, Mumbai, India; 3 G. I. Surgery, Seth GSMC & KEMH, Mumbai, India; 4 Department of Pathology, TNMC & Nair Ch hospital, Mumbai, India

**Keywords:** Hepatobiliary, Tuberculosis, Sclerosing cholangitis, Cholangiocarcinoma

## Abstract

**Background:**

Hepatobiliary tuberculosis includes miliary, tuberculous hepatitis or localized forms. The localised form is extremely uncommon and can mimic malignancy. Still rarer is its presentation as sclerosing cholangitis.

**Case presentation:**

A 50 year male presented with acute onset jaundice, significant weight loss and elevated liver enzymes with clinico-radiological suspicion of cholangiocarcinoma. A left hepatectomy was done and dilated bile ducts filled with caseous necrotic material were seen intra-operatively. Histopathology suggested localized hepatobiliary tuberculosis with features of secondary sclerosing cholangitis.

**Conclusion:**

Localised hepatobiliary tuberculosis can cause diagnostic difficulties and its possibility should be considered especially in endemic areas.

## Background

Tuberculosis (TB) is a worldwide health problem and an important cause of morbidity and mortality, especially in developing nations. While pulmonary involvement is commonest, extra-pulmonary TB is also an important clinical problem. Abdominal TB is one of the most prevalent forms with gastrointestinal, splenic, pancreatic, hepatobiliary, peritoneal, omental, mesenteric and/or abdominal lymph node involvement. Manifestations can be non-specific and mimic other conditions, including malignancies [1].

Sclerosing cholangitis is a chronic cholestatic disease, characterized by inflammation, obliterative fibrosis of bile ducts, stricture formation and progressive biliary destruction leading to cirrhosis. It occurs in two forms; primary/idiopathic (commonly) and acquired/secondary. The causes of latter include immune disorders, ischemia, infections, parasites, infiltrative processes and metastasis [2].

Here we report a case of localized hepatobiliary TB (HBTB) causing secondary sclerosing cholangitis (SSC), which is very uncommon.

## Case presentation

A 50 years male presented with jaundice since 10 days associated with significant loss of weight & appetite. Previous history of fever 1 month ago was present, for which he was diagnosed and treated as typhoid. He was treated for blood hypertension for 5 years with no previous history of surgery. There was no past history TB or TB contact. On examination, he was afebrile and icterus was present. Abdominal examination revealed soft, non-tender abdomen with no guarding, rigidity or organomegaly.

**Fig. 1:**
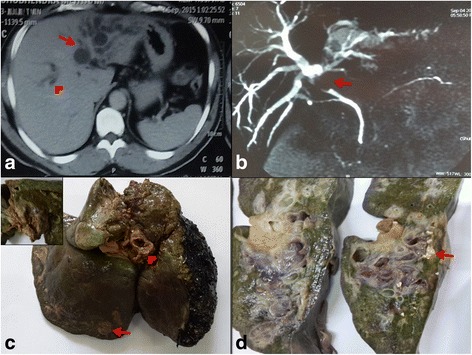
**a** CT scan showing dilatation of left intrahepatic bile duct (arrow), more than right (arrowhead); **b** MRCP showing filling defect (arrow) in common hepatic duct; **c** Left hepatectomy showing yellow-white nodules (arrow) with dilated, thickened hepatic ducts (arrowhead). Large thick walled bile duct showing necrotic material (Inset); **d** Multiple dilated, thick walled hepatic ducts, few studded with stones (arrow)

Hemogram was within normal. Sequential liver function tests were abnormal, with bilirubin values ranging 9–18.6 mg/dl, alkaline phosphatase 227-862 U/L, ALT 227-876 U/L and AST from 71 to 1043 U/L. AMA and viral markers (HBsAg/HCV/HCV/HAV/HIV) were negative. ECG and Chest X-ray were normal. Ultrasonography and CT scan showed mildly enlarged liver with asymmetric dilation of intra-hepatic bile duct and a proximal concentric ill-defined mildly enhancing soft tissue lesion suggesting cholangiocarcinoma [Fig. 1a]. Magnetic resonance cholangio-pancreatography (MRCP) showed filling defect in bile ducts likely to be calculus, cast or sludge along with few enlarged periportal lymph nodes [Fig. 1b].

Because fast progression of the disease, left hepatectomy with hepaticojejunostomy was done and during surgery, caseous necrotic material was seen oozing out from dilated bile ducts. Specimen measuring 12 × 12 × 6 cm showed dilated and thickened hepatic ducts, few studded with stones, along with 0.5-1 cm sized yellowish white nodules in the adjacent liver parenchyma [Fig. 1c, d]. Remaining parenchyma was non-cirrhotic with focal greenish discoloration.

**Fig. 2:**
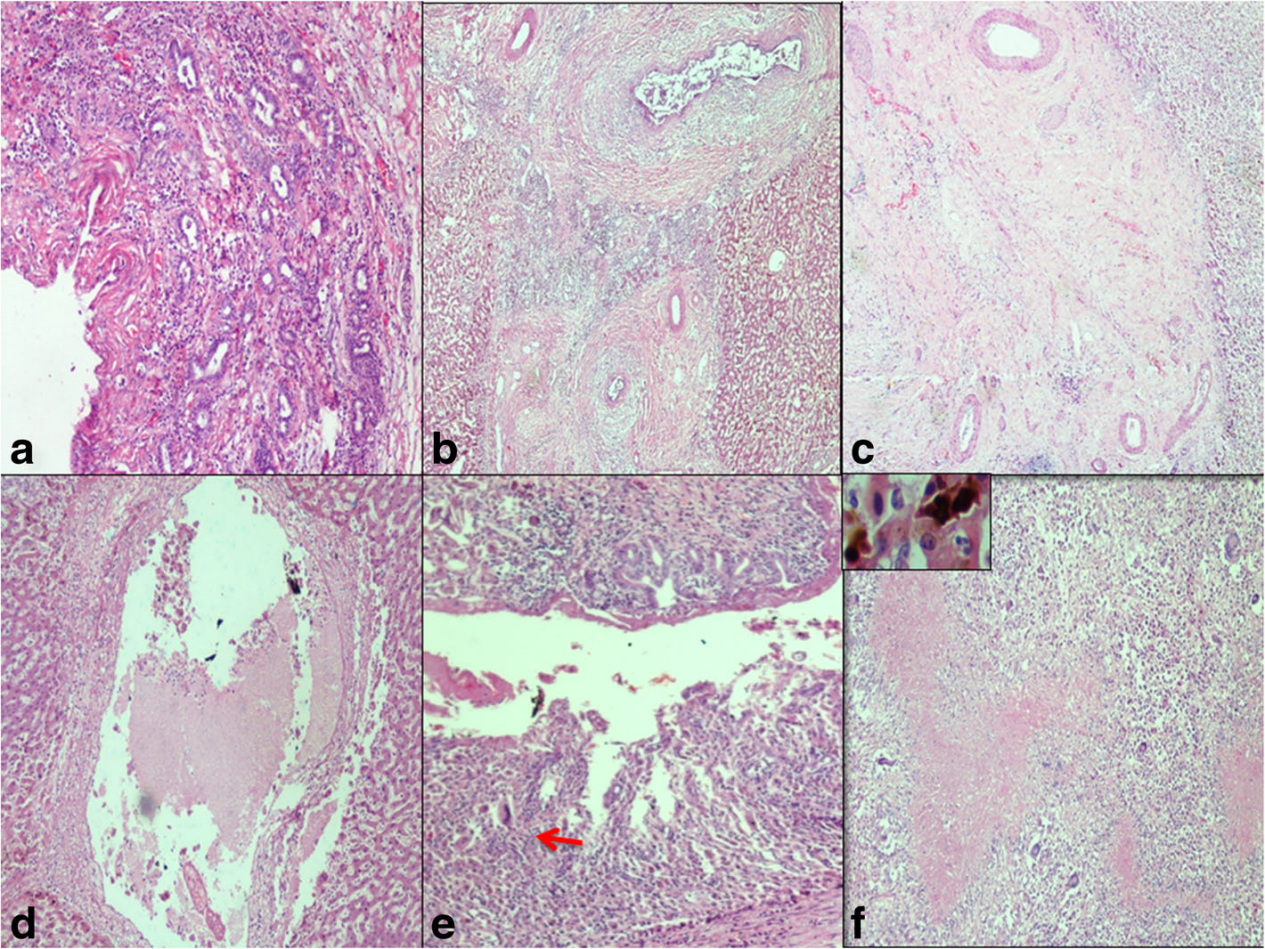
**a** Bile ductular proliferation; **b** Fibrous expansion of portal tracts with onion skinning of bile ducts; **c** Absent bile duct with collagen nodule; **d** Bile duct filled with caseous material; **e** Ulcerated bile duct with granuloma (arrow); **f** Caseating hepatic granulomas. Adjacent liver parenchyma showing cholestasis (Inset) (H & E,× 400)

On microscopy, fibrous expansion of portal tracts with bile ductular proliferation [Fig. 2a], extensive periductal fibrosis with focal onion skinning [Fig. 2b] and occasional collagen nodule formation was observed [Fig. 2c]. Bile ducts revealed caseous material in lumen with mucosal ulceration, occasional ill-defined granulomas and dense chronic inflammation composed of lymphoplasmacytic infiltrate with sheets of macrophages [Fig. 2d, e]. Liver parenchyma exhibited focal necrotising epithelioid granulomas and cholestasis [Fig. 2f]. Ziehl-Neelsen staining and PCR for acid-fast bacilli (AFB) and gomori-methanamine-silver for fungus were negative.

A diagnosis of localised HBTB with SSC-like features was made for which a trial of anti-tubercular treatment (ATT) was given. Patient responded well and PET-CT after treatment completion showed no residual fixation.

## Discussion

Liver is not fit for mycobacterium tuberculosis because of its low tissue oxygen tension. However, three types of HBTB have been defined: miliary, granulomatous disease (tuberculous hepatitis) and localised [3]. The commonest, generalized miliary, usually has no clinical features related to liver. Tuberculous hepatitis presents as fever, mild jaundice, with/without hepatomegaly and rare pulmonary involvement. The least common localised form, shows exclusive hepatobiliary tract involvement based on clinical, radiologic and laboratory examination, further confirmed on microbiology and histopathology [4]. Localised HBTB involves liver parenchyma, biliary tract or both with the latter two being rare even in endemic areas [4]. Ours was a case of localised HBTB with no gastrointestinal or pulmonary involvement.

Broadly, there are two forms of HBTB, diffuse and localised. The diffuse form occurs if the bacilli reach heaptic parenchyma via hepatic artery from lungs or through portal vein in case of gastrointestinal involvement. In localised form, bacilli may reach liver via lymphatics or through rupture of a tuberculous lymph node at porta [3, 5]. Biliary involvement can be secondary to AFB excretion into bile producing strictures or due to compression by enlarged nodes or hepatic granulomas, leading to obstructive jaundice [1]. In our case it was difficult to ascertain whether biliary involvement was secondary to hepatic parenchymal disease or due to enlarged periportal lymph nodes.

Bandopadhyay and Maithy have tabulated clinical features, laboratory abnormalities and outcome from seven large series of HBTB and observed that fever was the most common symptom (50–90%) followed by abdominal pain (45–66%). Other features were jaundice, pruritis, loss of weight and appetitie. Hepatomegaly was the commonest abnormality on clinical examination [3]. Our patient had obstructive jaundice, weight loss and mild hepatomegaly. Though there is no specific age for HBTB, most patients are between 30 and 50 years, similar to ours (50 years) [3, 4].

It is often difficult to differentiate benign and malignant causes of biliary stricture because clinical, laboratory findings including LFTs and cholangiography are non-specific [5]. Treatment HBTB is mainly medical and surgery may be required if there is biliary involvement. In our case, clinical features and CT suggested cholangiocarcinoma, left hepatectomy with hepatico-jejunostomy was indicated.

Grossly, liver can show nodules (tuberculoma/tubercular abscess) which may mimick primary or metastatic malignancy [1]. Biliary involvement can lead to diffuse thickening or strictures resembling primary sclerosing cholangitis or cholangiocarcinoma [5, 6]. In our case too, possibility of malignancy could not be ruled out on gross examination.

Microscopically, necrotizing caseating granulomas were observed in liver, which are considered pathognomic of TB. Bile ducts showed caseation in lumen, ulceration with dense chronic inflammation and vague granulomas in the wall. Caseating granulomas have also occasionally been reported in Hodgkin disease, brucellosis and few fungal infections like coccidioidomycosis, with different clinical presentations [3].

SSC aetiologies are various, including infection. In HBTB, SSC-like changes have been described on cholangiography [5, 6]. However in our case, similar changes were seen on histology, which have not been previously described. The other causes of SSC were excluded on clinico-radiological examination.

The positivity of AFB stains and cultures varies from to 45 and 60% respectively. Diaz et al. found that at least 57% of hepatic tuberculosis granulomas were positive on PCR [7]. In our study, AFB and PCR were negative which might be related to presence of only focal granulomas with paucity of mycobacteria in the tissue. Culture was not available because the diagnosis was not suspected preoperatively. Many clinicians accept good clinical response to anti-TB drugs is an indirect reliable tool for TB diagnosis. In our case too, patient responded dramatically to ATT supporting HBTB diagnosis.

## Conclusion

Atypical presentations of HBTB can lead to diagnostic difficulties; hence a high suspicion index is necessary for its diagnosis. Localised HBTB can cause SSC-like changes or stimulate malignant tumour.
